# Special Issue “Biomarkers and Early Detection Strategies of Ovarian Tumors”

**DOI:** 10.3390/ijms26189071

**Published:** 2025-09-18

**Authors:** Laura Aleksandra Szafron, Jolanta Kupryjanczyk, Lukasz Michal Szafron

**Affiliations:** 1 Department of Experimental Oncology, Maria Sklodowska-Curie National Research Institute of Oncology, 02-781 Warsaw, Poland; laura.szafron@gmail.com; 2 Department of Cancer Pathomorphology, Maria Sklodowska-Curie National Research Institute of Oncology, 02-781 Warsaw, Poland; jolanta.kupryjanczyk@nio.gov.pl

## 1. Introduction

Although progress has been made in developing new therapies and deepening the biological understanding of ovarian carcinoma (OvCa), it continues to be the most lethal gynecologic cancer in women. According to estimates from the American Cancer Society, approximately 20,890 new cases and 12,730 deaths from ovarian cancer are expected in the United States in 2025 [1]. Mortality rates are even higher in countries with limited cancer prevention, screening, and diagnostic programs. The poor prognosis of OvCa is largely due to the challenges of detecting the disease at an early, more treatable stage.

Ovarian carcinomas are classified into two major subtypes: high-grade (hgOvCa) and low-grade (lgOvCa). High-grade tumors are the predominant form and are marked by extensive genomic instability, chromosomal alterations, and frequent mutations in tumor suppressor genes such as *TP53*, *BRCA1*, and *BRCA2* [2]. In contrast, low-grade tumors are rare, typically diagnosed at a younger age, show relative resistance to chemotherapy, and are associated with longer survival. Unlike high-grade tumors, lgOvCa seldom harbor *TP53* or *BRCA1/2* mutations [3,4] and, particularly in the serous subtype, share molecular similarities with borderline ovarian tumors (BOTs) [5].

BOTs are uncommon tumors with low malignant potential, showing an intermediate characteristics between benign and invasive ovarian cancers. They generally arise in women of reproductive age, are diagnosed at early FIGO stages, and carry favorable survival rates. Preoperative imaging methods (ultrasound, MRI) aid in distinguishing BOTs from carcinomas, but definitive diagnosis requires histopathology. Surgical resection remains the primary treatment, with fertility-sparing approaches considered for younger patients desiring pregnancy. Chemotherapy, however, is not recommended [6,7]. Even after complete resection, about 20% of BOTs may recur—most as borderline tumors but, in some cases, as low-grade carcinomas [5,8,9,10].

While biomarkers in BOTs are poorly characterized, OvCa—particularly hgOvCa—has been extensively studied. Nonetheless, uncertainties persist regarding the clinical utility of some molecular markers. Thus, identifying reliable prognostic and predictive biomarkers remains critical for improving treatment outcomes and reducing OvCa-related mortality.

## 2. Ovarian Cancer Risk Factors

The most significant risk factors are inherited mutations in the *BRCA1* and *BRCA2* genes, which increase OvCa risk significantly, and have a strong influence on patient survival. Thus, such genetic alterations are considered a valuable stratification factor [11]. Consistently, mutations in other genes involved in homologous recombination repair, such as *RAD51C/D* and *BRIP1,* also elevate OvCa risk [12]. The higher OvCa risk is also observed in women, who have never been pregnant or have had fewer full-term pregnancies, likely due to uninterrupted (incessant) ovulation and hormonal exposure [13]. Early menarche and late menopause extend the number of ovulatory cycles, thus also raising OvCa risk [14]. Conversely, the use of combined oral contraceptives has been shown to reduce ovarian cancer risk, with longer use correlating with greater protection [15].

It is noteworthy that OvCa risk increases with aging and peaks between the ages of 50 and 80 years [16]. However, lifestyle factors, like inappropriate diet leading to obesity, may increase the risk of developing ovarian tumors. Obesity is associated with chronic inflammation and increased estrogen levels, which may promote tumor development. Moreover, some studies demonstrate that up to 40% of obese patients with ovarian cancer receive suboptimal doses of chemotherapy, which are not proportional to actual body weight, and such reduced dosage of chemotherapeutic agents may compromise progression-free survival (PFS) and overall survival (OS) [17].

Endometriosis and pelvic inflammatory disease are also linked to elevated ovarian cancer risk, however, some studies suggest that the cumulative incidence rate of ovarian cancer is significantly higher in patients with endometriosis than in those with pelvic inflammatory disease [18].

## 3. Prevention

Women with mutations in *BRCA1/2* are advised to undergo regular screening, including transvaginal ultrasound and CA-125 blood tests, although the effectiveness of screening alone is limited. Prophylactic removal of ovaries and fallopian tubes is currently the most effective preventive measure, significantly reducing the risk of ovarian cancer in *BRCA1/2*-mutation carriers [19].

Asymptomatic progression of OvCa underscores the urgent need for sensitive, minimally invasive diagnostic tools. One promising approach in recent years has been the use of circulating tumor DNA (ctDNA) as a biomarker for early cancer detection. ctDNA originates from fragments of DNA released by tumor cells into the bloodstream (and also to saliva, urine or cerebrospinal fluid). These fragments carry tumor-specific genetic mutations and epigenetic changes, providing a window into tumor biology through a simple blood draw—often termed a “liquid biopsy” [20]. Elevated ctDNA levels in OvCa patients correlate with poorer progression-free and overall survival [21]. Unlike traditional imaging techniques and CA-125 measurements, ctDNA enables real-time monitoring, detection of minimal residual disease, and earlier relapse identification. It was shown that after surgery the presence of ctDNA was a strong predictor of relapse (hazard ratio ~17.6), outperforming CA-125 [22]. Thus, while ctDNA-based screening for OvCa has neither been widely used in clinical practice nor approved by the American Food and Drug Administration (FDA) yet, it holds great promise for the diagnosis and recurrence monitoring of this neoplasm.

## 4. Diagnosis

The methods for ovarian cancer diagnosis include, e.g., the OVA1 test, which involves the assessment of five serum biomarkers—CA-125, tranthyretin, apolipoprotein A1, beta-2 microglobulin, and transferrin—into a single numerical score that reflects the malignant risk. OVA1 can detect malignancies (including early-stage ovarian cancers) that might be overlooked when evaluating CA-125 levels only [23]. Admittedly, the current guidelines from the Society of Gynecologic Oncology (SGO) do not recommend OVA1 as a standalone diagnostic or screening test for assessing adnexal masses preoperatively. Still, the SGO endorses OVA1 as an auxiliary tool in OvCa diagnosis [24].

## 5. Chemotherapy

Despite using the standard systemic chemotherapy for OvCa (typically a combination of platinum agents (like cisplatin or carboplatin) and taxanes (paclitaxel)), there has been growing interest in hyperthermic intraperitoneal chemotherapy (HIPEC). HIPEC involves perfusing the peritoneal cavity with heated chemotherapy immediately after cytoreductive surgery, aiming to eradicate microscopic residual disease. Hyperthermia enhances drug penetration and synergizes with platinum agents and taxanes, while limiting systemic toxicity. Additionally, hyperthermia has been shown to reduce the mechanisms of induced cellular resistance to cisplatin [25].

## 6. Targeted Therapy

Targeted therapies have become essential additions to standard platinum-taxane chemotherapy in the treatment of ovarian cancer. Among the most widely used are anti-angiogenic agents, Poly (ADP-Ribose) Polymerase (PARP) inhibitors or immune checkpoint inhibitors. Bevacizumab, a monoclonal antibody targeting VEGF-A, which inhibits angiogenesis, has demonstrated clinical usability in OvCa. The GOG-0218 and ICON7 clinical trials showed a significant PFS benefit when bevacizumab was added to standard chemotherapy [26].

Another monoclonal antibody, pembrolizumab, is an immune checkpoint inhibitor (it blocks the PD-1 receptor), which has been explored in platinum-resistant ovarian cancer. While the activity of pembrolizumab as a single-agent is modest, its combinations with bevacizumab and low-dose cyclophosphamide demonstrated clinical benefits in 25% of patients with recurrent OvCa [27].

PARP inhibitors (PARPi) exploit defects in DNA repair mechanisms, particularly in *BRCA1/2*-mutated and homologous recombination repair-deficient (HRD) tumors. Currently, four PARPi, olaparib, rucaparib, talazoparib and niraparib, are approved by regulatory agencies for the treatment of multiple tumor types including OvCa [28]. In patients with germline or somatic *BRCA1/2* mutations (which were deleterious or suspected to be deleterious), the olaparib monotherapy (SOLO1 trial) resulted in a three-year PFS benefit (HR, 0.30; 95% CI 0.23–0.41). On the other hand, the niraparib monotherapy in the PRIMA trial (which enrolled patients regardless of their *BRCA1/2* mutation status) demonstrated a median PFS benefit (HR, 0.40; 95% CI 0.27–0.62) [29].

## 7. Invitation for Paper Contribution

Given the considerations presented in this editorial, discovering novel molecular biomarkers that reflect qualitative or quantitative changes in the genomes, methylomes, transcriptomes, proteomes, or metabolomes of BOTs and OvCas is crucial in advancing the fight against these tumors. We hope that the findings shared in this Special Issue will lay the foundation for innovative, more effective, and less burdensome approaches to the detection, diagnosis, and treatment of ovarian neoplasms.

In order to make this Special Issue even more scientifically sound and interesting for the broader group of scientists, both clinicians and basic researchers, we would like to invite You to contribute a manuscript to this international endeavor (to date, six valuable research articles written by scientists from Japan, USA, Russia, Denmark, Switzerland, and Poland have been published). It is worth noting that both original and review articles are gladly welcome. If You wish to participate in this Special Issue by supporting it with Your knowledge and study results, we truly solicit Your involvement and warmly encourage You to submit Your manuscript by the deadline, i.e., 20 December 2025. We hope that this issue is likely to achieve another major goal, being its publication as a digital book available online, and that the precious contribution of You and all the other scientists involved will help us meet book-publishing requirements.

## References

[1] Ovarian Cancer Statistics|How Common Is Ovarian Cancer?. https://www.cancer.org/cancer/types/ovarian-cancer/about/key-statistics.html.

[2] Guo T., Dong X., Xie S., Zhang L., Zeng P., Zhang L. (2021). Cellular Mechanism of Gene Mutations and Potential Therapeutic Targets in Ovarian Cancer. Cancer Manag. Res..

[3] Babaier A., Mal H., Alselwi W., Ghatage P. (2022). Low-Grade Serous Carcinoma of the Ovary: The Current Status. Diagnostics.

[4] Wong K.-K., Bateman N.W., Ng C.W., Tsang Y.T.M., Sun C.S., Celestino J., Nguyen T.V., Malpica A., Hillman R.T., Zhang J. (2022). Integrated Multi-Omic Analysis of Low-Grade Ovarian Serous Carcinoma Collected from Short and Long-Term Survivors. J. Transl. Med..

[5] Hauptmann S., Friedrich K., Redline R., Avril S. (2017). Ovarian Borderline Tumors in the 2014 WHO Classification: Evolving Concepts and Diagnostic Criteria. Virchows Arch..

[6] Bourdel N., Huchon C., Abdel Wahab C., Azaïs H., Bendifallah S., Bolze P.-A., Brun J.-L., Canlorbe G., Chauvet P., Chereau E. (2021). Borderline Ovarian Tumors: French Guidelines from the CNGOF. Part 2. Surgical Management, Follow-up, Hormone Replacement Therapy, Fertility Management and Preservation. J. Gynecol. Obstet. Hum. Reprod..

[7] Timor-Tritsch I.E., Foley C.E., Brandon C., Yoon E., Ciaffarrano J., Monteagudo A., Mittal K., Boyd L. (2019). New Sonographic Marker of Borderline Ovarian Tumor: Microcystic Pattern of Papillae and Solid Components. Ultrasound Obstet. Gynecol..

[8] Niu L., Tian H., Xu Y., Cao J., Zhang X., Zhang J., Hou J., Lv W., Wang J., Xin L. (2021). Recurrence Characteristics and Clinicopathological Results of Borderline Ovarian Tumors. BMC Women’s Health.

[9] Shih K., Zhou Q., Huh J., Morgan J., Iasonos A., Aghajanian C., Chi D., Barakat R., Abu-Rustum N. (2011). Risk Factors for Recurrence of Ovarian Borderline Tumors. Gynecol. Oncol..

[10] Gershenson D.M., Silva E.G., Levy L., Burke T.W., Wolf J.K., Tornos C. (1998). Ovarian Serous Borderline Tumors with Invasive Peritoneal Implants. Cancer.

[11] Alsop K., Fereday S., Meldrum C., deFazio A., Emmanuel C., George J., Dobrovic A., Birrer M.J., Webb P.M., Stewart C. (2012). BRCA Mutation Frequency and Patterns of Treatment Response in BRCA Mutation-Positive Women with Ovarian Cancer: A Report from the Australian Ovarian Cancer Study Group. J. Clin. Oncol..

[12] Suszynska M., Ratajska M., Kozlowski P. (2020). BRIP1, RAD51C, and RAD51D Mutations Are Associated with High Susceptibility to Ovarian Cancer: Mutation Prevalence and Precise Risk Estimates Based on a Pooled Analysis of ~30,000 Cases. J. Ovarian Res..

[13] Husby A., Wohlfahrt J., Melbye M. (2022). Pregnancy Duration and Ovarian Cancer Risk: A 50-Year Nationwide Cohort Study. Int. J. Cancer.

[14] Pięta B., Chmaj-Wierzchowska K., Opala T. (2012). Past Obstetric History and Risk of Ovarian Cancer. Ann. Agric. Environ. Med..

[15] Arshadi M., Hesari E., Ahmadinezhad M., Yekta E.M., Ebrahimi F., Azizi H., Esfarjani S.V., Rostami M., Khodamoradi F. (2024). The Association between Oral Contraceptive Pills and Ovarian Cancer Risk: A Systematic Review and Meta-Analysis. Bull. Cancer.

[16] Zheng G., Yu H., Kanerva A., Försti A., Sundquist K., Hemminki K. (2018). Familial Risks of Ovarian Cancer by Age at Diagnosis, Proband Type and Histology. PLoS ONE.

[17] Benedetto C., Salvagno F., Canuto E.M., Gennarelli G. (2015). Obesity and Female Malignancies. Best Pract. Res. Clin. Obstet. Gynaecol..

[18] Huang J.-Y., Yang S.-F., Wu P.-J., Wang C.-H., Tang C.-H., Wang P.-H. (2021). Different Influences of Endometriosis and Pelvic Inflammatory Disease on the Occurrence of Ovarian Cancer. Int. J. Environ. Res. Public Health.

[19] Gadducci A., Sergiampietri C., Tana R. (2013). Alternatives to Risk-Reducing Surgery for Ovarian Cancer. Ann. Oncol..

[20] Golara A., Kozłowski M., Cymbaluk-Płoska A. (2024). The Role of Circulating Tumor DNA in Ovarian Cancer. Cancers.

[21] Taliento C., Morciano G., Nero C., Froyman W., Vizzielli G., Pavone M., Salvioli S., Tormen M., Fiorica F., Scutiero G. (2024). Circulating Tumor DNA as a Biomarker for Predicting Progression-Free Survival and Overall Survival in Patients with Epithelial Ovarian Cancer: A Systematic Review and Meta-Analysis. Int. J. Gynecol. Cancer.

[22] Hou J.Y., Chapman J.S., Kalashnikova E., Pierson W., Smith-McCune K., Pineda G., Vattakalam R.M., Ross A., Mills M., Suarez C.J. (2022). Circulating Tumor DNA Monitoring for Early Recurrence Detection in Epithelial Ovarian Cancer. Gynecol. Oncol..

[23] Dunton C.J., Hutchcraft M.L., Bullock R.G., Northrop L.E., Ueland F.R. (2021). Salvaging Detection of Early-Stage Ovarian Malignancies When CA125 Is Not Informative. Diagnostics.

[24] SGO Position Statement: OVA-1 Society of Gynecologic Oncology. https://www.sgo.org/news/sgo-position-statement-ova-1/.

[25] Riggs M.J., Pandalai P.K., Kim J., Dietrich C.S. (2020). Hyperthermic Intraperitoneal Chemotherapy in Ovarian Cancer. Diagnostics.

[26] Babaier A., Ghatage P. (2023). Among Patients with Advanced Ovarian Carcinoma, Who Benefits from Bevacizumab the Most?. Ann. Transl. Med..

[27] Zsiros E., Lynam S., Attwood K.M., Wang C., Chilakapati S., Gomez E.C., Liu S., Akers S., Lele S., Frederick P.J. (2021). Efficacy and Safety of Pembrolizumab in Combination With Bevacizumab and Oral Metronomic Cyclophosphamide in the Treatment of Recurrent Ovarian Cancer: A Phase 2 Nonrandomized Clinical Trial. JAMA Oncol..

[28] Herzog T.J., Vergote I., Gomella L.G., Milenkova T., French T., Tonikian R., Poehlein C., Hussain M. (2023). Testing for Homologous Recombination Repair or Homologous Recombination Deficiency for Poly (ADP-Ribose) Polymerase Inhibitors: A Current Perspective. Eur. J. Cancer.

[29] Kim J.H., Kim S.I., Park E.Y., Kim E.T., Kim H., Kim S., Park S.-Y., Lim M.C. (2024). Comparison of Survival Outcomes between Olaparib and Niraparib Maintenance Therapy in BRCA-Mutated, Newly Diagnosed Advanced Ovarian Cancer. Gynecol. Oncol..

